# Characterizing the evolution life cycle of the Sunkoshi landslide in Nepal with multi-source SAR data

**DOI:** 10.1038/s41598-020-75002-y

**Published:** 2020-10-22

**Authors:** Meng Ao, Lu Zhang, Yuting Dong, Lijun Su, Xuguo Shi, Timo Balz, Mingsheng Liao

**Affiliations:** 1 State Key Laboratory of Information Engineering in Surveying, Mapping, and Remote Sensing, Wuhan University, Wuhan, China; 2 Faculty of Information Engineering, China University of Geosciences, Wuhan, China; 3 Key Laboratory for Mountain Hazards and Earth Surface Process of CAS, Institute of Mountain Hazards and Environment, Chinese Academy of Sciences, Chengdu, China

**Keywords:** Natural hazards, Imaging and sensing

## Abstract

A catastrophic landslide disaster happened on 2 August 2014 on the right bank of Sunkoshi River in Nepal, resulting in enormous casualties and severe damages of the Araniko highway. We collected multi-source synthetic aperture radar (SAR) data to investigate the evolution life cycle of the Sunkoshi landslide. Firstly, Distributed Scatterers SAR Interferometry (DS-InSAR) technology is applied to analyze 20 ALOS PALSAR images to retrieve pre-disaster time-series deformation. The results show that the upper part, especially the top of the landslide, has long been active before collapse, with the largest annual LOS deformation rate more than − 30 mm/year. Time series deformations measured illustrate that rainfall might be a key driving factor. Next, two pairs of TerraSAR-X/TanDEM-X bistatic data are processed to identify the landslide affected area by intensity change detection, and to generate pre- and post-disaster DSMs. Surface height change map showed maximum values of − 150.47 m at the source region and 55.65 m in the deposit region, leading to a debris volume of 5.4785 ± 0.6687 million m^3^. Finally, 11 ALOS-2 PALSAR-2 and 82 Sentinel-1 SAR images are analyzed to derive post-disaster annual deformation rate and long time series displacements of the Sunkoshi landslide. The results illustrated that the upper part of the landslide were still in active deformation with the largest LOS displacement velocity exceeding − 100 mm/year.

## Introduction

Landslide disasters along highway in mountainous area are increasing progressively to constitute a majority of highway disaster events^1^. Many landslides show characteristics of large scale, complex mechanism and catastrophic losses. It has become a great challenge for scientists and engineers of geotechnical engineering to understand and mitigate landslide hazards in mountainous areas. For this purpose, the spatial–temporal evolution pattern of deformation field for a certain landslide during its life cycle should be well known. Unfortunately, such information is usually difficult to obtain using traditional ground-based measurements that are only available at sparse points. As a modern geodetic technology, synthetic aperture radar interferometry (InSAR) can be utilized to solve above problem and has been successfully applied in landslide monitoring^2–5^. However, most landslide investigations focus on a specific stage only, such as pre-disaster deformation signal extraction^6–10^, quantification of mass wasting volume^11,12^ and post-failure displacement monitoring^13,14^, while there has been few application of InSAR to comprehensively characterize a collapsed landslide during its pre-, co- and post-disaster stages.

**Figure 1:**
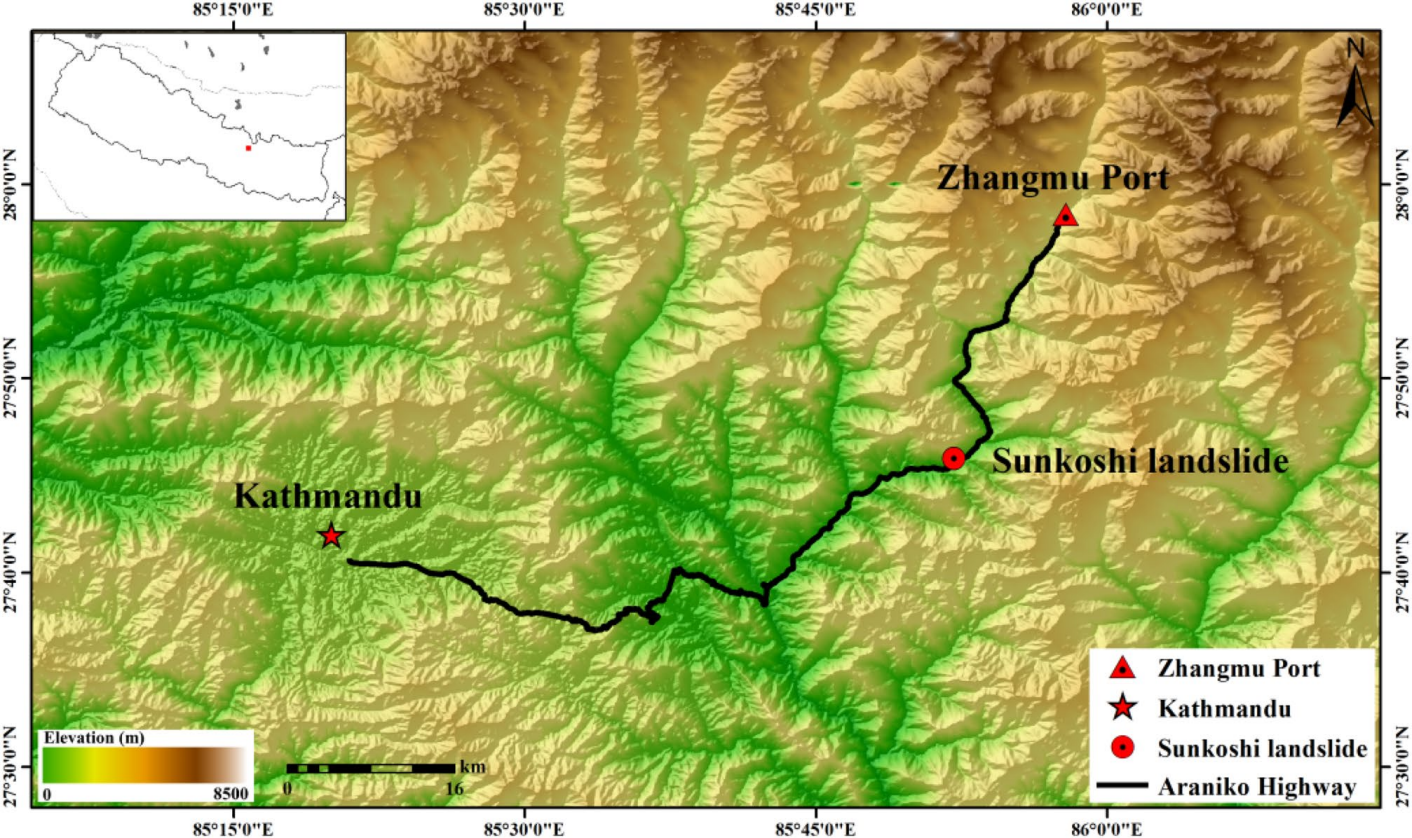
Location of the Sunkoshi landslide along with the Araniko Highway. The subfigure in the upper-left corner shows the relative position of Sunkoshi landslide (red rectangle) in Nepal. The map was generated using ESRI ArcGIS 10.1 (https://www.esri.com/en-us/home).

The Sunkoshi landslide is located at the northern half of the Araniko highway. The road was built in 1960s with the assistance of China, with the total span of about 115 km from the Zhangmu Port on the border between China and Nepal to Kathmandu, the capital of Nepal (as shown in Fig. 1). It is not only the main trade and personnel channel connecting China and Nepal, but also the lifeline of rescue and the major transport artery for the local earthquake stricken areas.

The highway passes through the contact zone between Indian plate and Eurasian plate that is raised along with continuing Himalayan orogeny induced by inter-plate collision and compression^15^, with an average uplifting range of more than 3000 m. It is one of the most active areas of tectonic movement on the Earth. The strong neotectonic movement makes the bedrock part metamorphosed and broken, providing abundant loose solid source for landslide activity.

Along the highway, the height difference between the mountain peak and the valley floor is up to several thousand meters, forming natural steep slopes more than 30° with some part nearly vertical. This area is also characterized by complex geologic, hydrologic and climatic conditions, as well as frequent earthquake disasters. In particular, the Mw 7.8 Gorkha earthquake that happened on 25 April 2015 caused strong ground motions and triggered thousands of landslides in the exceedingly steep topography of Nepal^16–21^. Since the Quaternary, landslides, collapses, rock piles and debris flows are extremely developed in Nepal, which has brought adverse effects on the implementation and protection of the highway.

Affected by strong and concentrated precipitation during the Indian monsoon season, the annual precipitation in many areas can reach 2500 mm^15^. In the rainy season, with the special topography of the landslide area, the surface water cannot be discharged along the surface runoff in time, but fully infiltrates along the surface cracks and loose block gravel soil layer. On one hand, it makes the sliding body heavier and increases the sliding force. On the other hand, the weak soil layer will become further saturated and softened, and the strength reduced rapidly. Rainfall caused events of landslides, debris flows and other natural disasters often happened, which seriously affected the post-disaster reconstruction and the daily life of local people.

The Sunkoshi landslide happened on 2 August 2014 at the K83 mileage of Araniko highway, which caused severe casualties (at least 156 deaths) and destroyed the highway of about 700 m. As a result, the operation of the Araniko highway has been suspended for a long period, which brought great difficulties to goods transportation and economic communication along the line, and also hampered the vigorous development of the golden tourism route between China and Nepal. The Sunkoshi River flowing through the slope toe was blocked by slipped rocks, forming a dam of about 56 m high and a large volume barrier lake. Supplementary Fig. S1 online shows multi-temporal panoramic images from Google Earth that can reflect the evolution of the Sunkoshi landslide. The instability of landslide and the barrier lake posed great threats upon the operation of the downstream hydropower station as well as human life and property safety.

Although the collapse of Sunkoshi landslide had a strong impact on the Sino-Nepal transportation and the Nepal social-economic development, more attentions have been paid to the safety of dam and barrier lake formed by the landslide^22–25^, while very few scientific investigations of this landslide stability analysis has been carried out, which may be primarily due to its remote location and low accessibility.

## Results

In this study, the evolution life cycle of the Sunkoshi landslide during different periods (pre-, co- and post-disaster stages) is characterized using various InSAR techniques with multi-source SAR data. The deformation pattern and possible driving factors in the pre-disaster stage are explored, the sliding area is determined with the collapse volume estimated, and the post-disaster stability of the landslide is evaluated. Such kinds of information should be of significance to help decision making for post-disaster treatment and risk management.

### Retrieval of pre-disaster time-series deformations

The Sunkoshi landslide is located in the northern mountainous area of Nepal with steep topography, complex geologic background and dense vegetation. With a long wavelength, ALOS PALSAR data can maintain high coherence and achieve deep penetration into vegetation. 20 scenes of ALOS PALSAR images are analyzed to inverse the LOS surface deformation. The SRTM DEM of 3 arc-second resolution is used for the estimation and removal of flat-earth and topographic phase components. Both Distributed Scatterer SAR Interferometry (DS-InSAR) and Small Baseline Subset SAR Interferometry (SBAS-InSAR) techniques are applied to retrieve time-series displacements. The SBAS-InSAR functionality was fully implemented in the StaMPS software package, while the DS-InSAR processing chain was established in a mixed way by self-developing the preprocessing steps and adopting the spatial–temporal 3D phase unwrapping procedure embedded in StaMPS.

**Figure 2:**
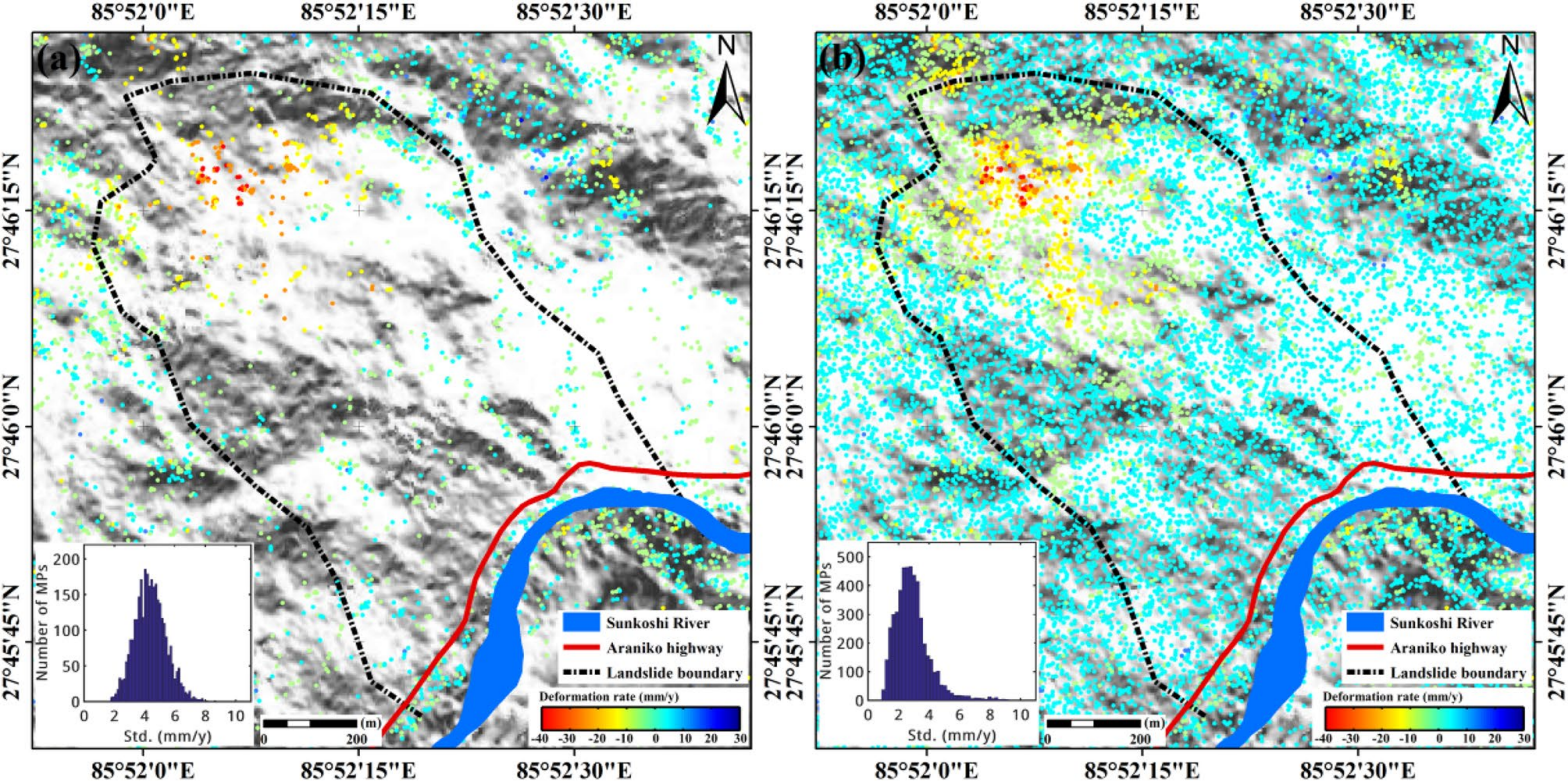
Results of pre-disaster annual deformation rate of Sunkoshi landslide obtained by (**a**) SBAS-InSAR and (**b**) DS-InSAR, separately. The Sunkoshi River and Araniko highway are rendered in their original locations before destruction. The subfigures on the lower-left corner show the histograms of standard deviation distribution of deformation rate. The maps were generated using ESRI ArcGIS 10.1 (https://www.esri.com/en-us/home).

Firstly, annual mean LOS deformation rates of Sunkoshi landslide during pre-disaster stage derived by SBAS-InSAR and DS-InSAR separately are shown in Fig. 2a,b. The landslide surface before collapse was largely covered by vegetation, leading to very sparse measurement points (MPs) identified in the SBAS result. The spatial density of MPs detected by DS-InSAR is much higher than that of SBAS by about 13.5 times. With significantly increased number of MPs, more detailed information on the spatial pattern of the landslide surface deformation is clearly presented. We can observe in Fig. 2b that obvious instability appeared in the upper part of the landslide, with the largest deformation rate higher than − 30 mm/year at the crown, while the lower part of the slope was relatively stable without evident sign of movement. The entire landslide mass can be divided into two zones with one resistant layer in between. The upper part of the landslide is steep with an average slope of 52.45°, while the lower part is relative gentle with a mean slope of 37.44°. Such a terrain condition made the upper part prone to failure, and might be a contribution factor leading to the collapse of the landslide in August 2014.

**Figure 3:**
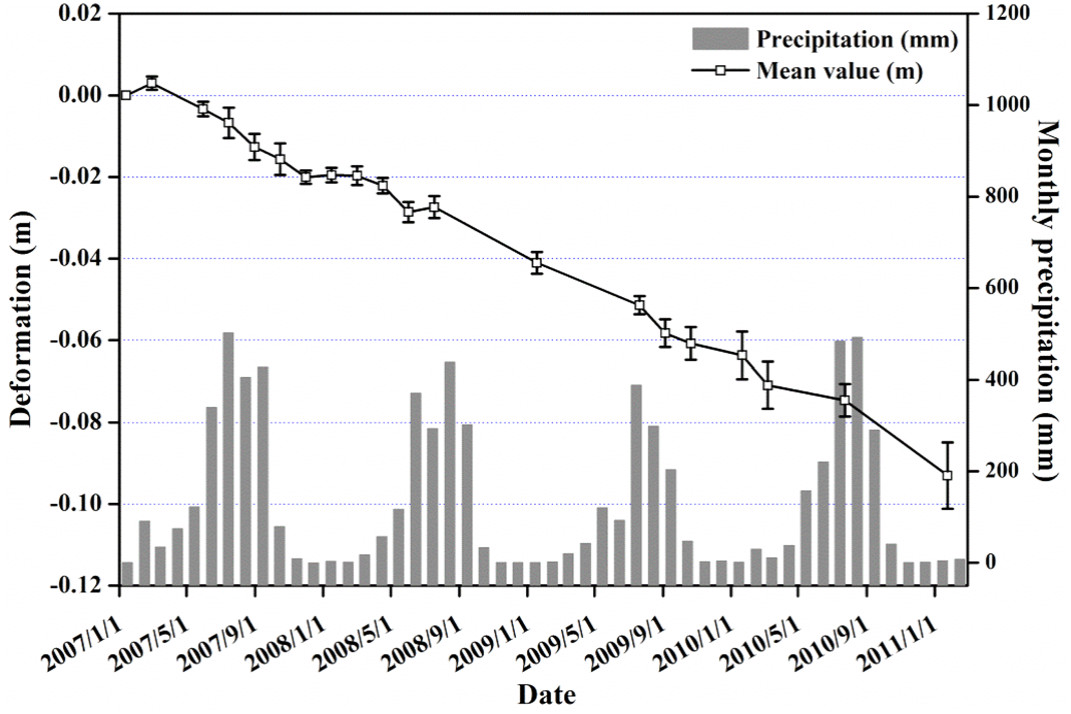
Pre-disaster time-series deformation of Sunkoshi landslide. Grey bar chart illustrates the monthly cumulative rainfall. White squares connected by the black lines represent the time-series measurements of annual mean LOS displacements of MPs on the landslide crown, with the vertical error bars indicating standard deviation or uncertainties of measurements.

The climate of Nepal belongs to a typical monsoon climate type. In its northern mountainous area characterized by humid with mist clouds, and the precipitation is abundant in rainy season. We used the archived precipitation data over this area acquired by the NASA Tropical Rainfall Measuring Mission (TRMM) to verify whether the historic deformations of the Sunkoshi landslide were correlated with seasonal rainfall. The time series mean cumulative displacements of MPs within the deforming region (the top part of the landslide) as well as the precipitation data are plotted in Fig. 3.

We can see that the annual precipitation was mainly concentrated in summer from June to September. The deformation shows an overall linear trend with seasonal variations that might be related to rainfall. The maximum cumulative deformation in four years was more than − 100 mm. During the months of heavy rainfall, acceleration of the deformation tended to show up. Moreover, the landslide disaster occurred on 2 August 2014, hence it is reasonable to identify rainfall as an important factor that can deeply affect the landslide stability. However, more information on the geological structure and deformation mechanism of the landslide are needed to further validate if precipitation is the main cause of the 2014 collapse event.

### Slope surface changes observed from TSX/TDX bistatic data pairs

After the landslide disaster happened, the surface underwent dramatic changes due to collapse and sliding, leading to serious decorrelation. Two pairs of TerraSAR-X/TanDEM-X (TSX/TDX) bistatic SAR data acquired before and after the 2014 Sunkoshi landslide event were processed to generate pre- and post-disaster DSMs to further quantify the slope surface changes. Landslide-affected area can be identified by intensity change detection, and the debris volume can be estimated through surface height change mapping.

#### Change detection with SAR backscatter intensity

In this section, the approach of intensity differencing between pre- and post-disaster SAR images is employed to identify the failure-affected area. Firstly, SAR amplitude images showing radar backscatter intensity are multi-looked to suppress the speckles. Then subpixel level geometric alignment of multitemporal SAR images is carried out to facilitate change detection.

**Figure 4:**
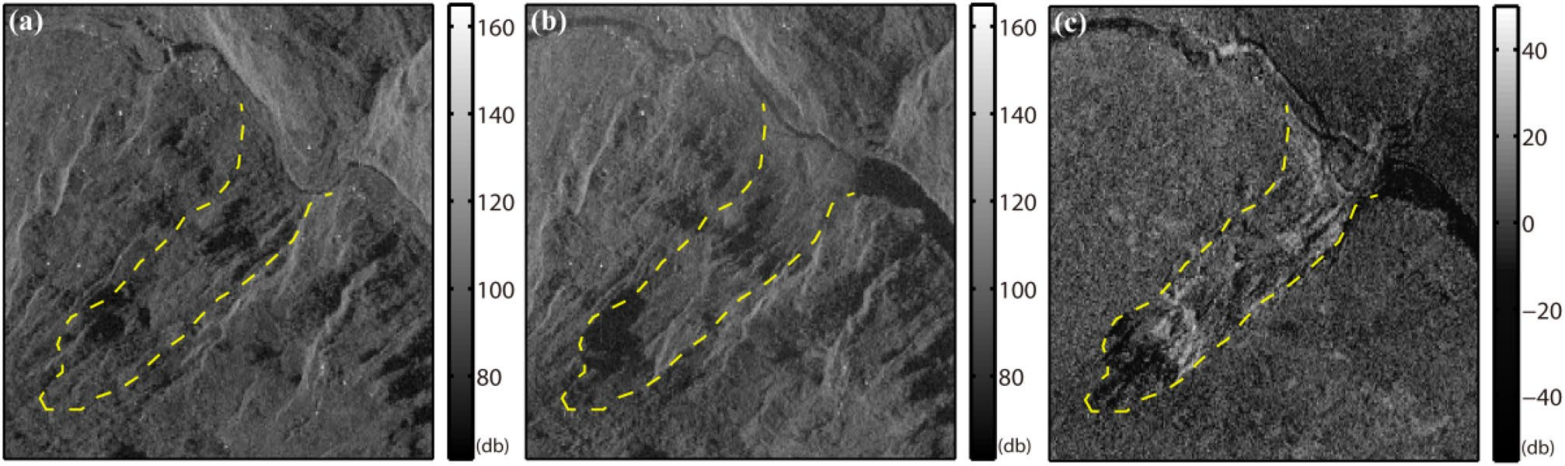
Intensity map of (**a**) TDX 20130307 before and (**b**) TDX 20140908 after collapse as well as (**c**) intensity difference between two TDX SAR intensity rendered in radar coordinate system. Logarithmic transformation is applied to convert intensity values into decibel (dB) expressions. The dashed yellow line outlines the landslide boundary.

Figure 4 shows the intensity map before (a) and after (b) collapse, with the intensity difference in (c). The landslide formed barrier lake can be easily identified in Fig. 4b. The river channel beneath the landslide was reshaped from a curve into a straight line after dredging the river blockage induced by debris flow. In Fig. 4c, dense vegetation and shrubs were demolished and moved away, and rocks and soils were exposed, resulting in the intensity brightening. The landslide crown was cut off, yielding a steeper slope, and the nearly vertical landslide trailing edge can be seen from Fig. 8a to be shown later, which corresponds to the obviously large shadow area in Fig. 4b.

The mean intensities of 20 ALOS PALSAR images and 11 ALOS-2 PALSAR-2 images are also produced and rendered in Supplementary Fig. S2 online. We can clearly identify similar changes in backscattering characteristics of the landslide surface before and after the collapse as shown in Fig. 4.

The difference of intensity reflects the temporal alteration of backscattering characteristics of the ground surface, which can be applied to identify the landslide affected area, but it cannot quantify the actual variation of the surface landscape.

#### Estimation of slope surface height changes

**Figure 5:**
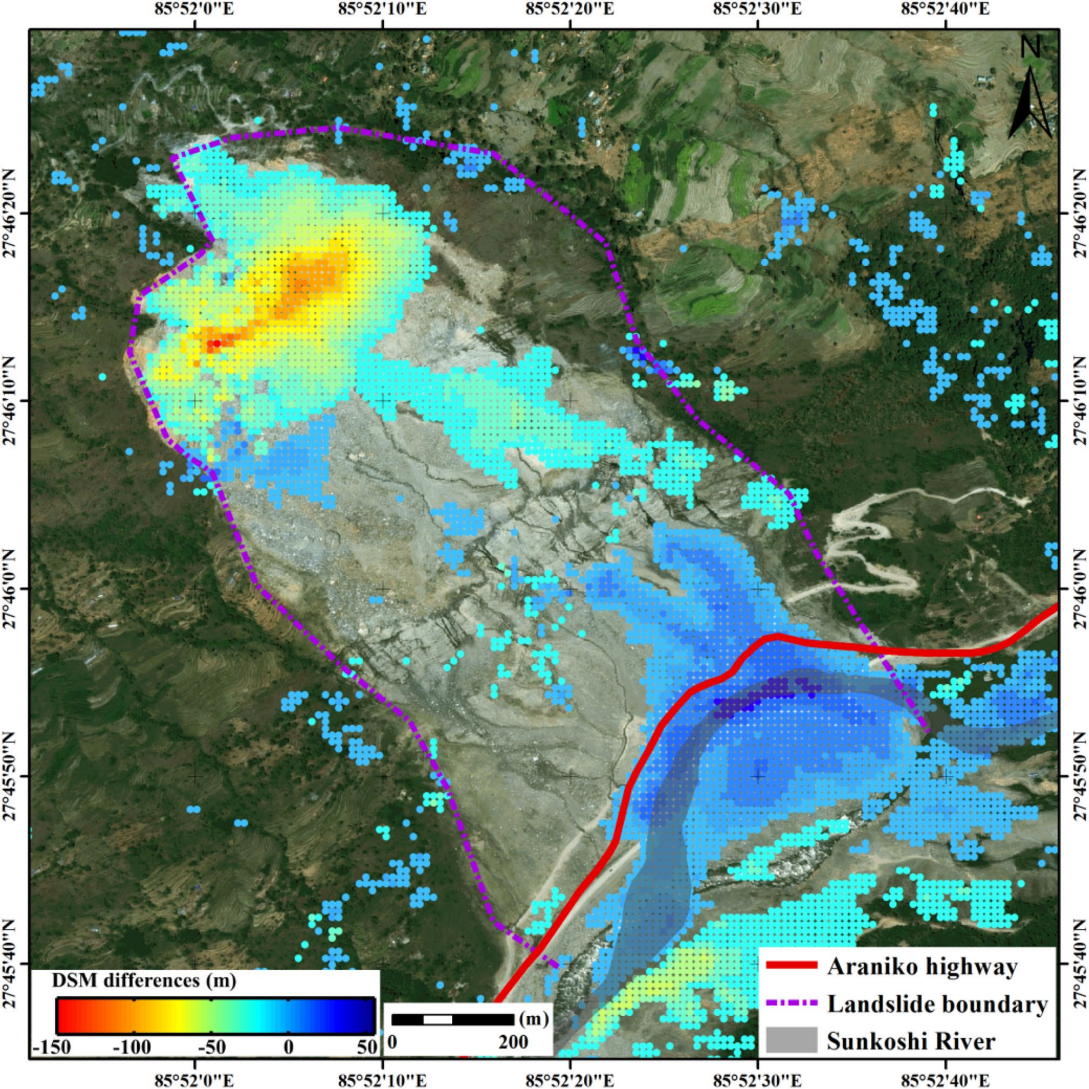
Elevation change map generated by differencing the pre- and post-disaster DSMs. Resolution cells with height differences less than 10 m are masked out. The map was generated using ESRI ArcGIS 10.1 (https://www.esri.com/en-us/home).

In order to quantitatively assess the surface damage caused by the collapse, the height change map is generated by differentiating the pre- and post-disaster DSMs, as shown in Fig. 5. It can be divided into three zones: the depletion zone on the top, the scraping zone in the middle, and the accumulation zone at the slope toe. The maximum change of surface height reached − 150.47 m at the depletion zone and 55.65 m in the accumulation zone. The depletion zone with the maximum height difference is exactly the same as the area with the largest deformation rate before collapse, while the accumulation zone where elevation changed remarkably is just located at the course of Sunkoshi River, and the elevation difference indicates the height of the landslide dam.

**Figure 6:**
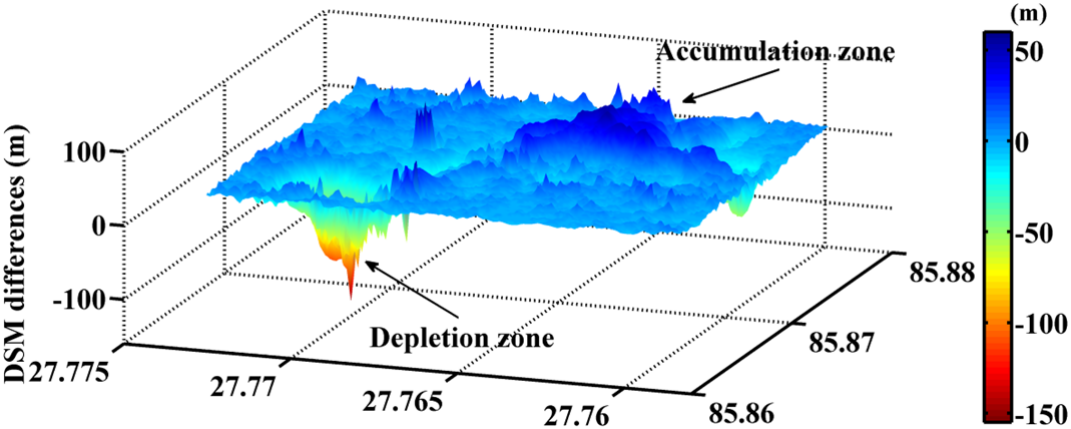
Three dimensional map of the height differences used for calculating the debris volume. The map was generated using Matlab R2014.

A large volume of mass wasting destroyed the road and diverted the river to form a huge barrier lake. The mass volume collapsed from the depletion zone is estimated from the height difference map (as shown in Fig. 6). Tandem-X DEMs have a relative vertical accuracy of 4 m for areas with a slope greater than 20%^26^, and a theoretically synthetic uncertainty of 5.6569 m can be produced in height differences between pre- and post-disaster DSMs. By multiplying the vertical uncertainty with the area of depletion zone, we can roughly assess the uncertainty of mass volume estimation. As the result, the total debris volume of the Sunkoshi landslide was estimated as 5.4785 ± 0.6687 million m^3^.

### Post-disaster deformation mapping

After the collapse event in August 2014, the road was dredged and the barrier lake was discharged. As the Araniko highway is the primary transportation channel linking China and Nepal, the unstable Sunkoshi landslide still poses long-term threats on human and vehicles passing by, hence post-disaster deformation monitoring is a crucial task to prevent possible catastrophic events in near future. In consideration of SAR data availability, we divided the post-disaster stage into two overlapping periods, i.e. period I from September 2014 to October 2017, and period II from January 2017 to October 2019.

Eleven ALOS-2 PALSAR-2 images were collected to investigate deformations of the Sunkoshi landslide during period I. Eighteen interferograms generated from ALOS-2 data pairs with good coherence were chosen to perform Stacking InSAR analysis. The post-disaster DEM derived from TSX/TDX bi-static data pair was utilized to remove the flat-earth and topographic phase components. In addition, the phase ramp, baseline errors and residual terrain errors are estimated and removed from the unwrapped phase to enable multi-interferogram stacking.

**Figure 7:**
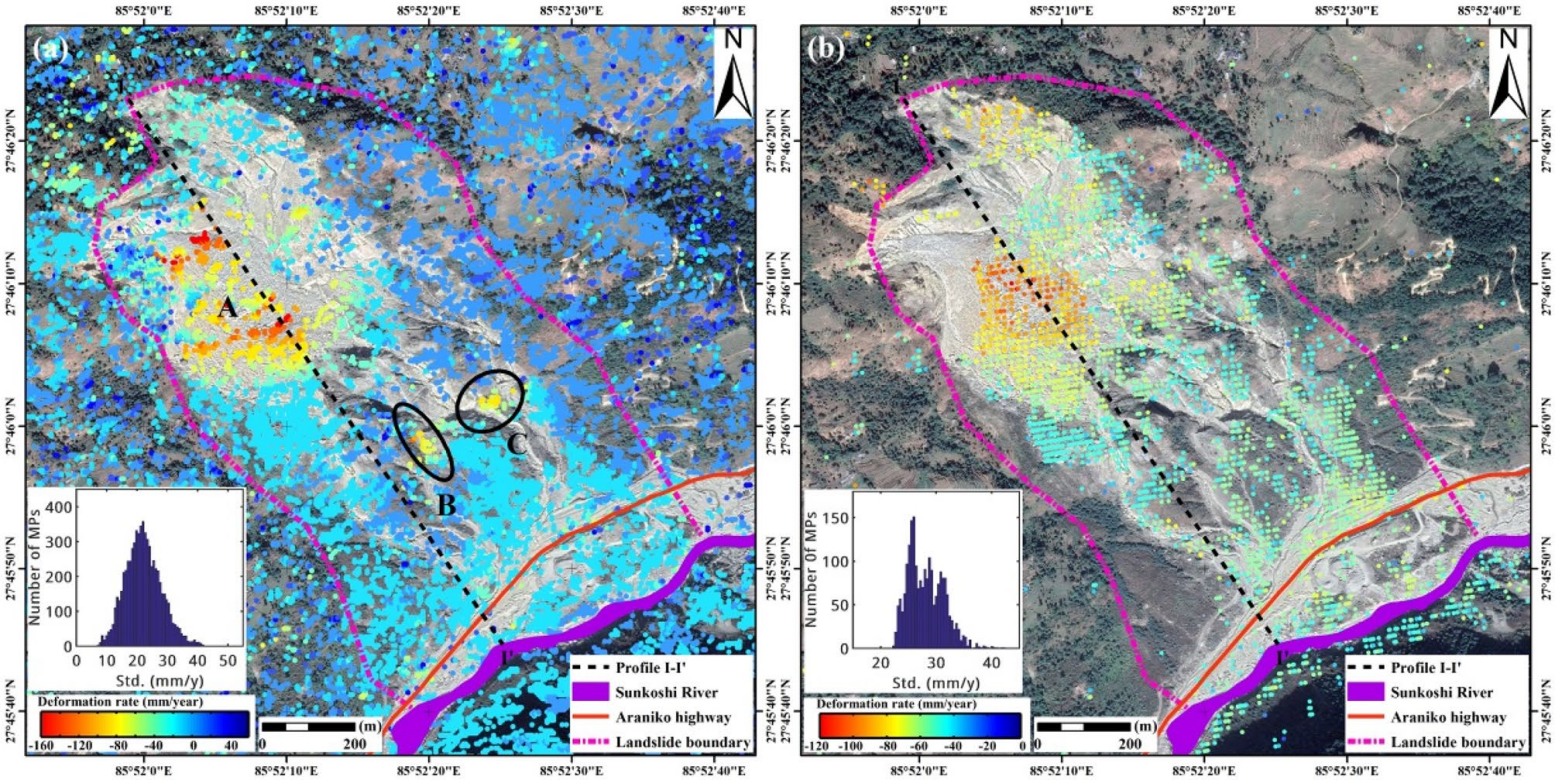
Post-disaster annual mean LOS deformation rate of Sunkoshi landslide measured by (**a**) ALOS-2 data during period I and (**b**) Sentinel-1 data during period II. The repaired road and dredged river are also rendered. The subfigure in the lower-left corner shows the histogram of standard deviation distribution of deformation rate. The map was generated using ESRI ArcGIS 10.1 (https://www.esri.com/en-us/home).

The annual mean deformation rate of Sunkoshi landslide during period I is illustrated in Fig. 7a. The upper part of the landslide was still deforming, and the largest deformation rate exceeded − 140 mm/year. The result shows that a large volume of loose debris was accumulated on the resistant layer in the upper part of the landslide (region A), which was very unstable and prone to slide in case of heavy rainfall, and thus may cause secondary disaster. In addition, significant deformations appeared at another two unstable spots (circles B and C) in the lower part of the landslide where the debris was accumulated.

**Figure 8:**
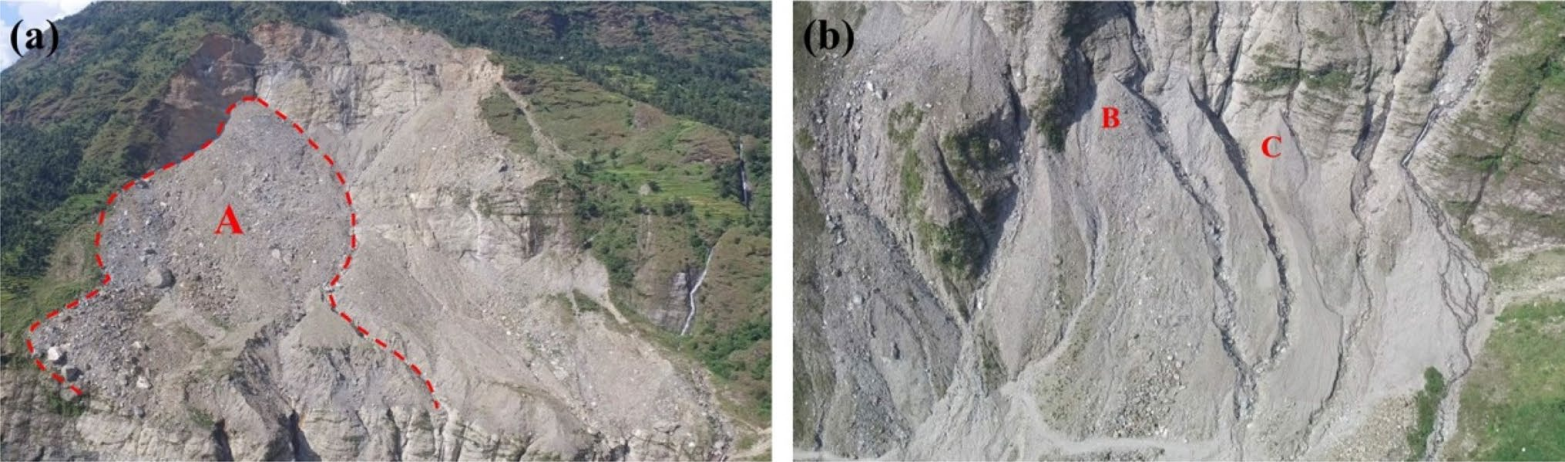
Photos of deformed debris flow accumulation areas after collapse. (**a**) Main deformation area (area A) in the upper part of landslide. (**b**) Unstable spots (area B and C) in the lower part. Photos were taken by Dr. Lijun Su.

The in-situ photos taken in October 2015 are given in Fig. 8. The steep rock wall induced by the collapse at the top and the resistant layer in the middle of the landslide can be clearly identified. In addition, the debris flow generated by the slide was accumulated in the upper and lower parts of the landslide. Specifically, Fig. 8a shows the area in the most active deformation (area A), while areas B and C shown in Fig. 8b are another two unstable spots with loose structure at the lower part.

The last acquisition of ALOS-2 PALSAR-2 data we obtained was on 28 October 2017. In order to make clear the current state of the Sunkoshi landslide, eighty-two Sentinel-1 SAR images were analyzed to measure the more recent deformations of the Sunkoshi landslide during period II. The result is shown in Fig. 7b. Due to the limited resolution of Sentinel-1 image and the problem of temporal decorrelation for C-band InSAR in vegetated area, sparse MPs are identified on the rock-exposed landslide body, while there is almost no MP in the surrounding vegetated areas. Similar to the results derived from ALOS-2 data, clear deformation signal appeared in the area of debris accumulation in the upper part of the landslide with the largest deformation rate as high as − 120 mm/year. In addition, although the diversion road around the Sunkoshi landslide has been put into operation and the traffic was flowing again on the Araniko highway, instability was found at the landslide toe around the reopened highway due to the destruction of accumulated debris by road dredging and heavy rainfall, as shown in Supplementary Fig. S3 online.

**Figure 9:**
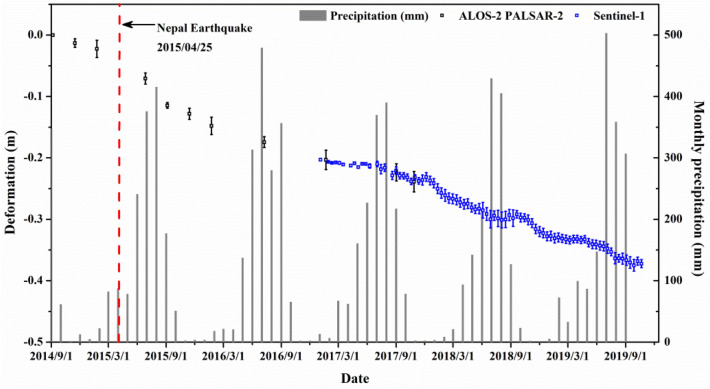
Long time-series deformation of region A. The dashed red line marks the date of Gorkha Earthquake happened on 25April 2015.

Long time-series displacements at MPs within region A extracted from ALOS-2 PALSAR-2 and Sentinel-1 data stacks are plotted in Fig. 9, in which the date of Mw 7.8 Gorkha Earthquake (25 April 2015) is marked by the vertical dashed red line. A study employed offset tracking with ALOS-2 data to measure the co-seismic landslide displacements along the Araniko highway caused by the devastating 2015 Gorkha earthquake, and their results revealed that the largest surface displacements were 2 m in azimuth and 1.2 m in range direction, respectively^27^. Therefore, it is necessary to investigate the post-seismic landslide deformation to evaluate the impact of earthquake on the long-term landslide stability.

An obvious acceleration after the earthquake can be observed from Fig. 9 in spite of the sparse measurements due to the limited number of ALOS-2 images. Afterwards, the deformation has become slowing down gradually to evolve into a linear pattern since early 2016, particularly demonstrated by the dense time series displacements measured by Sentinel-1 data. Nevertheless, the maximum cumulative deformation during the past five years has reached over − 400 mm. Furthermore, there seems no significant dependence between landslide deformation and rainfall, which might be primarily due to lack of sufficient ALOS-2 observations during period I.

The mean deformation rates over pre- and post-disaster stages together with the elevations of SRTM and post-disaster TanDEM are extracted along the profile I–I′ (marked in Fig. 7a) and plotted in Supplementary Fig. S4 online. Most debris detached from the source area was accumulated at the slope toe. The elevation change in the accumulation zone was identified as wide-spreading and slight to moderate increase. The post-disaster deformation detected in the accumulation area was relatively stable. In contrast, a large volume of debris was accumulated on the resistant layer in the upper part of the landslide. Since such debris was loosely structured without any stabilization, this elevated area could be in high risk of catastrophic sliding in cases of earthquake or heavy rainfall.

## Discussion

Landslides are a kind of geohazard widely spread in the northern mountainous area of Nepal. As this area is located in the active Hymalayan tectonic belt with summer monsoon climate, these landslides are vulnerable to events of earthquake and heavy rainfall. Although the population density in the northern Nepal is relatively low, those landslides close to human settlements and major transportation links still pose great threats upon local people and passengers. The Sunkoshi landslide investigated here is a typical example.

Wide-area detection, regular monitoring and disaster early warning are crucial tasks for the efficient management of landslide hazards to prevent and mitigate possible catastrophic slope failure events. Interferometry and pixel offset tracking (POT) with repeat-pass satellite SAR observations can be employed as powerful geodetic tools as they are capable of measuring land surface displacements that range from millimeters to meters. In particular, traditional two-pass DInSAR and Stacking InSAR techniques are usually first applied to carry out preliminary detection of potential landslides across wide areas. However, there might be big uncertainty in their results due to decorrelations and atmospheric disturbances.

Methods of time series InSAR analysis including PSI, SBAS-InSAR, and DS-InSAR, etc., need a long stack of repeat-pass SAR data as input. They can be used to not only derive more accurate results of landslide detection than DInSAR, but also perform regular monitoring of long-term landslide surface displacements. Generally speaking, the conventional PSI method is much less applicable than SBAS-InSAR and DS-InSAR in mountainous areas of Nepal.

Two fundamental products can be derived by time series InSAR analyses. One is the mean LOS displacement rate map showing the spatial pattern of deformations. The other is the time series displacements of MPs that can reveal temporal evolution of deformations. Dramatic accelerations in time series displacements might be taken as a kind of precursory signal of possible forthcoming slope failures to facilitate landslide disaster early warning, which has been demonstrated by several studies using long time series Sentinel-1 datasets^28,29^.

An additional lesson we can learn from the case study of the Sunkoshi landslide is that it is necessary to evaluate post-disaster slope stability by continuing deformation monitoring after the collapse, because the remaining part of the slope as well as the accumulated debris might be still in high risk of secondary failure subject to triggering factors such as earthquakes and intensive rainfall. The measurements of post-disaster deformation can support decision making on disaster relief and following treatments to avoid further damages and losses.

Multi-source SAR datasets are used in the case study of the Sunkoshi landslide. X-band high-resolution TerraSAR-X/TanDEM-X bistatic data pairs are specifically used to reconstruct pre- and post-disaster slope topography for quantitative identification of slope surface changes as well as estimation of collapsed mass volume. Archived L-band ALOS PALSAR data (2007–2011), L-band ALOS-2 PALSAR-2 data (2014–2017) and C-band Sentinel-1 data (2017–2019) are analyzed by DS-InSAR, Stacking InSAR and SBAS-InSAR techniques respectively to retrieve landslide surface displacements during the pre- and post-disaster stages. Comparisons among their results suggested that L-band data with longer wavelength and higher resolution outperforms C-band data in characterizing spatial pattern of deformation, while C-band data of much higher acquisition frequency shows advantage of revealing more detailed temporal evolution pattern of deformations.

Due to the limitation of SAR data availability and revisit period, most landslide monitoring investigations are focusing on a specific stage and cannot reveal the whole evolution process of the landslides. Therefore, the primary objective of this study is to make full use of archived and in orbit satellite data and employ various InSAR techniques to characterize the life cycle of the Sunkoshi landslide, i.e. including exploration of deformation pattern and possible driving factors in the pre-disaster stage, identification of the sliding area as well as estimation of the collapsed mass volume, and evaluation of the post-disaster slope stability. The results can provide important information to support deformation mechanism analysis of the Sunkoshi landslide and enable secondary disaster prevention.

## Conclusion

As a typical example of catastrophic landslide disasters along the Araniko highway in north Nepal, little detail on the historic evolution of the Sunkoshi landslide has been known till now due to its remote location and absence of ground measurement. In this study, we analyzed multi-source satellite SAR datasets to characterize the evolution life cycle of this landslide. Our major findings are summarized as follows.

First, according to the results of time series InSAR analyses with ALOS PALSAR data, the upper part of the landslide has long become unstable before collapse, with the maximum mean LOS displacement rate of about − 30 mm/year. Joint analysis of time series displacements retrieved and monthly cumulative precipitation records suggested that rainfall was the predominant impact factor for the landslide stability, and the 2014 failure event might be triggered by intensive rainfall in the summer monsoon season.

Second, by differencing pre- and post-disaster DSMs generated from two pairs of bistatic mode TerraSAR-X/TanDEM-X data over the Sunkoshi landslide, we quantified the slope surface height changes induced by the collapse event and estimated the collapsed mass volume. The results show that the maximum surface height changes reached − 150.47 m at the depletion zone and 55.65 m in the accumulation zone, leading to a debris volume of 5.4785 ± 0.6687 million m^3^.

Thirdly, we evaluated the post-disaster slope stability of the Sunkoshi landslide with InSAR analyses of ALOS-2 PALSAR-2 and Sentinel-1 data. The results clearly indicated that the upper part of the landslide was still in active deformation since late 2014 and thus the potential risk of catastrophic failure in the future cannot be ignored. Furthermore, intermittent accelerations of displacement can be observed, which might be induced by the 2015 Gorkha earthquake and heavy rainfall of summer monsoon. Therefore, regular monitoring of the slope stability with operational SAR data acquisitions should be carried out to facilitate landslide risk management and disaster early warning.

## Methods

### DS-InSAR processing

The Sunkoshi landslide is located at a high-altitude area with dense vegetation and complicated topography. Traditional InSAR technologies cannot identify sufficient measurement points and the low spatial density of detectable coherent points generally results in phase unwrapping errors and consequently big uncertainties in the deformation measurements^30^. Aiming at this problem, DS-InSAR is applied to ensure high spatial density of MPs extracted from the ALOS PALSAR data stack, and to solve the difficult problem of mapping landslide surface deformation in mountainous areas. Two key processing steps for DS-InSAR analysis are DS target identification and optimal phase estimation.

#### DS identification

DS is the coherent ensemble of radar echo signals from all small scatterers within the same resolution cell, among which none of these scatterers is dominant^31^, and they behave as statistically homogenous pixels (SHP). K-S test is usually used to calculate and select the appropriate DS by testing if the amplitude time series of two pixels follow the same probability distribution function^32^, which can be intrinsically formulated as a nonparametric goodness-of-fit test^33^. In this study, those pixels centered at a window of fixed size 25 × 25 with more than 20 SHPs are selected as DS candidates. The resultant SHP map for the landslide region outlined in Supplementary Fig. S5(a) online is shown in Supplementary Fig. S5(b) online. We can see clearly that the number of SHPs identified strongly depends on the local topography. Specifically, a large number of SHPs are detected over homogenous areas on the hillside, while for targets along stream channels in valleys the numbers of SHPs are much lower.

#### Optimal phase estimation

For the identified SHP of pixel *P*, the sample coherence matrix can be estimated by^32^:

1 $$ \Gamma (P) = \frac{1}{\left| \Omega \right|}\sum\limits_{P \in \Omega } {d^{\prime}(P) \cdot d^{{\prime}{\text{H}}} } (P) $$

where ^H^ indicates Hermitian conjugation and $\Omega$ represents a homogeneous group containing SHP pixels. $d^{\prime}(P)$ is the normalized complex reflectivity vector.

The optimal phase series ${{\varvec{\uptheta}}} = \left[ {\theta_{1} ,\theta_{2} ,\ldots,\theta_{N} } \right]^{{\text{T}}}$ of each pixel can be estimated by the maximum likelihood estimation (MLE), the first value of ${\varvec{\theta}}$ is set as zero, i.e. $\theta_{1} = 0$:

2 $$ \theta_{{{\text{ML}}}} = \mathop {\arg \max }\limits_{\uptheta } \{ {\varvec{\eta}}^{{\text{H}}} ( - |\Gamma |{^\circ }\Gamma ){\varvec{\eta}}\} $$

where ${\varvec{\eta}} = [0,e^{{j\theta_{2} }} , \ldots e^{{j\theta_{N} }} ]^{{\text{T}}}$ and the symbol ° represents the mathematic operator of Hadamard product between two matrices. Phase linking approach is applied to solve this equation quite efficiently in closed form as^34^:

3 $$ \hat{\theta }_{n}^{k} = \arg \left\{ {\sum\limits_{m \ne n}^{N} {\{ |\Gamma |^{ - 1} \}_{mn} \{ \Gamma \}_{mn} \exp (j\hat{\theta }_{m}^{k - 1} )} } \right\} $$

where *k* is the iteration step and the starting point of the iteration was assumed as the phase of the vector ${\varvec{\eta}}_{0} = 1$.

The DS candidates identified are then combined together with PS candidates to establish the observation network, upon which the standard PS-InSAR algorithm implemented in the StaMPS software package is applied to estimate the deformation time series of each MP^35–37^. The processing flowchart is shown in Supplementary Fig. S6 online.

### Surface change analyses with TSX/TDX bistatic SAR data pairs

After landslide disaster, the slope surface will undergo dramatic changes, leading to serious decorrelations. We make use of SAR intensity information to detect the landslide affected area^38^ with multitemporal SAR images acquired over the same area. In consideration of dissimilar imaging geometries adopted by SAR observations of multiple epochs, geometric correction and image registration are needed before performing change detection.

Surface height changes can be deduced by differencing two digital surface models (DSMs) acquired at two different dates^39^. For this purpose, two pairs of TSX/TDX bistatic SAR data were processed to generate pre- and post-disaster DSMs representing the surface topography before and after the collapse. Then a DSM change map was created to evaluate the mass volume of debris accumulated.

### Post-disaster stacking InSAR and SBAS-InSAR analysis

A limited number of 11 ALOS-2 PALSAR-2 images acquired after the collapse event were collected. It is usually difficult to apply time-series InSAR methods to achieve reliable results from such a small and temporarily sparse group of SAR observations. Therefore, stacking InSAR technique is adopted to retrieve post-disaster deformation during period I.

In this study, the post-disaster DSM derived from TSX/TDX bi-static data pair was utilized to remove flat-earth and topographic phase components from eighteen high-quality interferograms generated with ALOS-2 data pairs. Then all selected *n* interferograms with good coherence generated from *N* SAR images are unwrapped after proper corrections of phase ramp, baseline errors and residual height errors. With the assumptions of almost linear deformation pattern and random atmospheric disturbances, the deformation rate of the study area can be estimated as below according to the stacking InSAR technique^40^:

$$ V_{{{\text{stacking}}}} = - \frac{\lambda }{4\pi }\frac{{\sum\limits_{i = 1}^{n} {\varphi_{i} } }}{{\sum\limits_{i = 1}^{n} {tb_{i} } }} $$

where $\varphi_{i}$ is the unwrapped phase measurement of the *i*th interferogram, and *tb*_*i*_ is the corresponding temporal baseline.

In consideration of the strong temporal decorrelation for C-band InSAR in vegetated area and the high computational cost of the DS-InSAR method, we employed the SBAS-InSAR technique^41^ to evaluate the landslide stability with 82 Sentinel-1 SAR images. 240 differential interferograms were generated following the criteria of small spatial and temporal baselines (here the upper limits are 200 m for spatial baseline and 120 days for temporal baseline). The quantitative relationship between the unknown mean phase velocity $v$ and the unwrapped interferometric phase observation *δφ* can be expressed as:

$$ Bv = \delta \varphi $$

$$ v^{{\text{T}}} = \left[ {v_{1} = \frac{{\varphi_{1} }}{{t_{1} - t_{0} }}, \ldots ,v_{N} = \frac{{\varphi_{N} - \varphi_{N - 1} }}{{t_{N} - t_{N - 1} }}} \right] $$

where $\delta \varphi^{{\text{T}}} = \left[ {\delta \varphi_{1} , \ldots ,\delta \varphi_{M} } \right]$ is the vector of unwrapped phases of *M* interferograms generated from *N* + 1 SAR images acquired at epochs *t*_0_ to *t*_*N*_. *B* is the coefficient matrix of size *M* × *N* recording temporal baselines for these interferograms. The unknown parameters of deformation velocities as well as time series deformations can be derived by singular value decomposition (SVD) and least squares estimation^41^.

### SAR datasets

We acquired 20 scenes of ALOS PALSAR images from January 2007 to January 2011, 11 scenes of ALOS-2 PALSAR-2 images from September 2014 to October 2017, 82 scenes of Sentinel-1 SAR images from 4 January 2017 to 21 October 2019, and two pairs of TerraSAR-X/TanDEM-X (TSX/TDX) bistatic SAR data acquired before and after the 2014 Sunkoshi landslide event. Supplementary Table S1 online lists the acquisition parameters of all the datasets. Approximate ground coverage of all above SAR datasets are shown in Supplementary Fig. S7 online. Their spatial and temporal distributions are illustrated in Supplementary Fig. S8 online.

## Supplementary information

Supplementary information.

## Data Availability

The datasets generated and/or analyzed during the current study are available from the corresponding author on reasonable request.
