# Supplementary material for: Characterizing the evolution life cycle of the Sunkoshi landslide in Nepal with multi-source SAR data
Source: Sci Rep. 2020 Oct 22;10:17988. doi: 10.1038/s41598-020-75002-y (PMC7582190; doi:10.1038/s41598-020-75002-y)
Supplement: Supplementary file 1 — Supplementary information. [file 41598_2020_75002_MOESM1_ESM.docx]

**Characterizing the evolution life cycle of the Sunkoshi landslide in Nepal with multi-source SAR data**

Meng Ao, Lu Zhang, Yuting Dong, Lijun Su, Xuguo Shi, Timo Balz, Mingsheng Liao

**Supplementary materials**


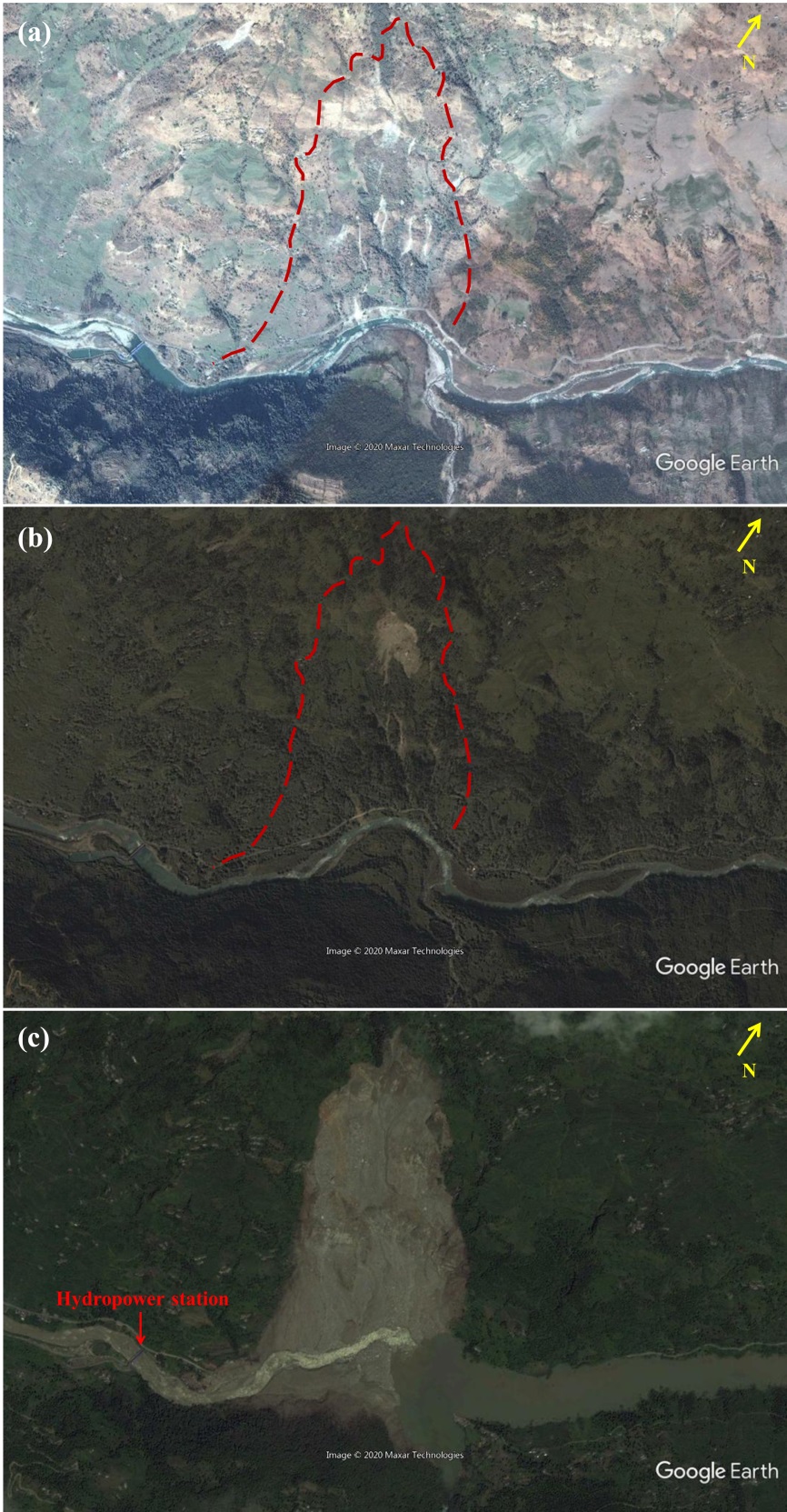


**Supplementary Fig. S1**. Google Earth panoramic images of the Sunkoshi landslide acquired on (a) February 18, 2009 and (b) October 6, 2012 before collapse, and (c) August 10, 2014 after collapse (eight days after the landslide disaster).

Supplementary Fig. S1 online shows multi-temporal panoramic images from Google Earth that can roughly reflect the evolution of the Sunkoshi landslide. We can observe the freshly fractured rock and the flow of water over the surface in the upper scarp in (a) that was acquired five years before the collapse. In the image of 2012 shown in (b), a small-scale rock slope failure with loose and unstable colluvium piled on the upper slope, as well as obvious gullies in the lower part can be seen clearly. In (c) showing the image taken shortly after the disaster event, we can see the huge exposed landslide body (1,200 m long in south-north direction, 700 m wide in east-west direction) and the barrier lake. The occurrence of the collapse event coincided with the rainy season and the river water level rose. The catchment length of the barrier lake reached 2.4 km and the catchment area is about 0.51 km^2^. Moreover, the Sunkoshi Hydropower Station was just one kilometer away from the barrier lake, as marked by the red arrow in (c).


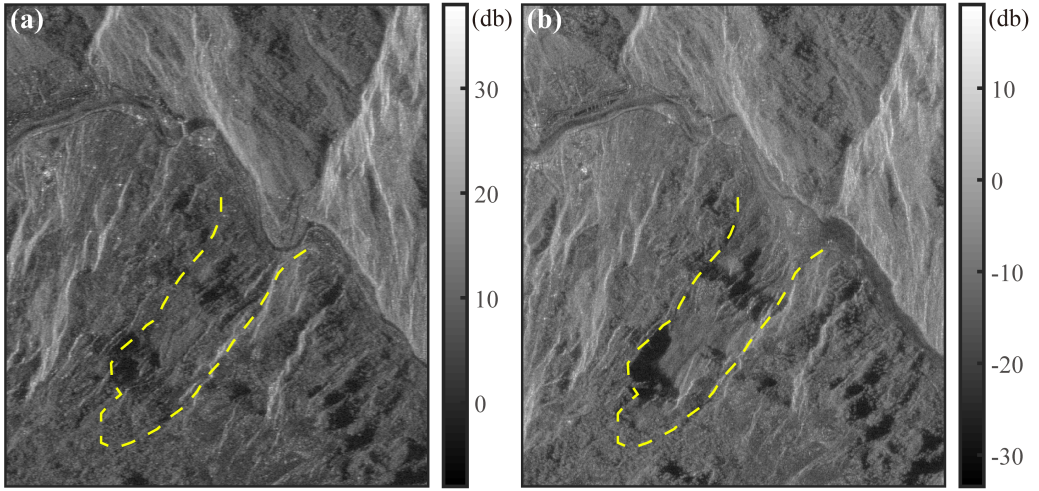


**Supplementary Fig. S2**. Mean intensity maps of (a) ALOS PALSAR and (a) ALOS-2 PALSAR-2 image stacks.

The mean intensities of 20 ALOS PALSAR images and 11 ALOS-2 PALSAR-2 images are produced and rendered in Supplementary Fig. S2 online.


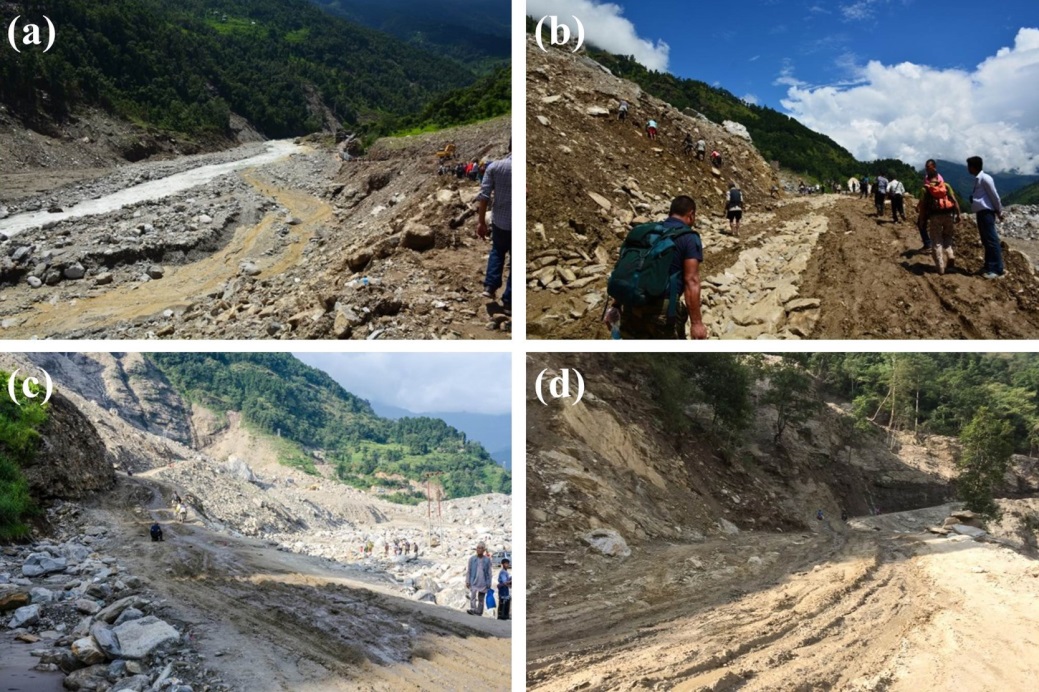


**Supplementary Fig. S3**. Photos of the reopened Araniko highway. Photos were provided by AGU Landslide Blog (http://blogs.agu.org/landslideblog/).

Although the diversion road around the Sunkoshi landslide has been put into operation and the traffic was flowing again on the Araniko highway, instability was found at the landslide toe around the reopened highway due to the destruction of accumulated debris by road dredging and heavy rainfall, as shown in Supplementary Fig. S3 online.


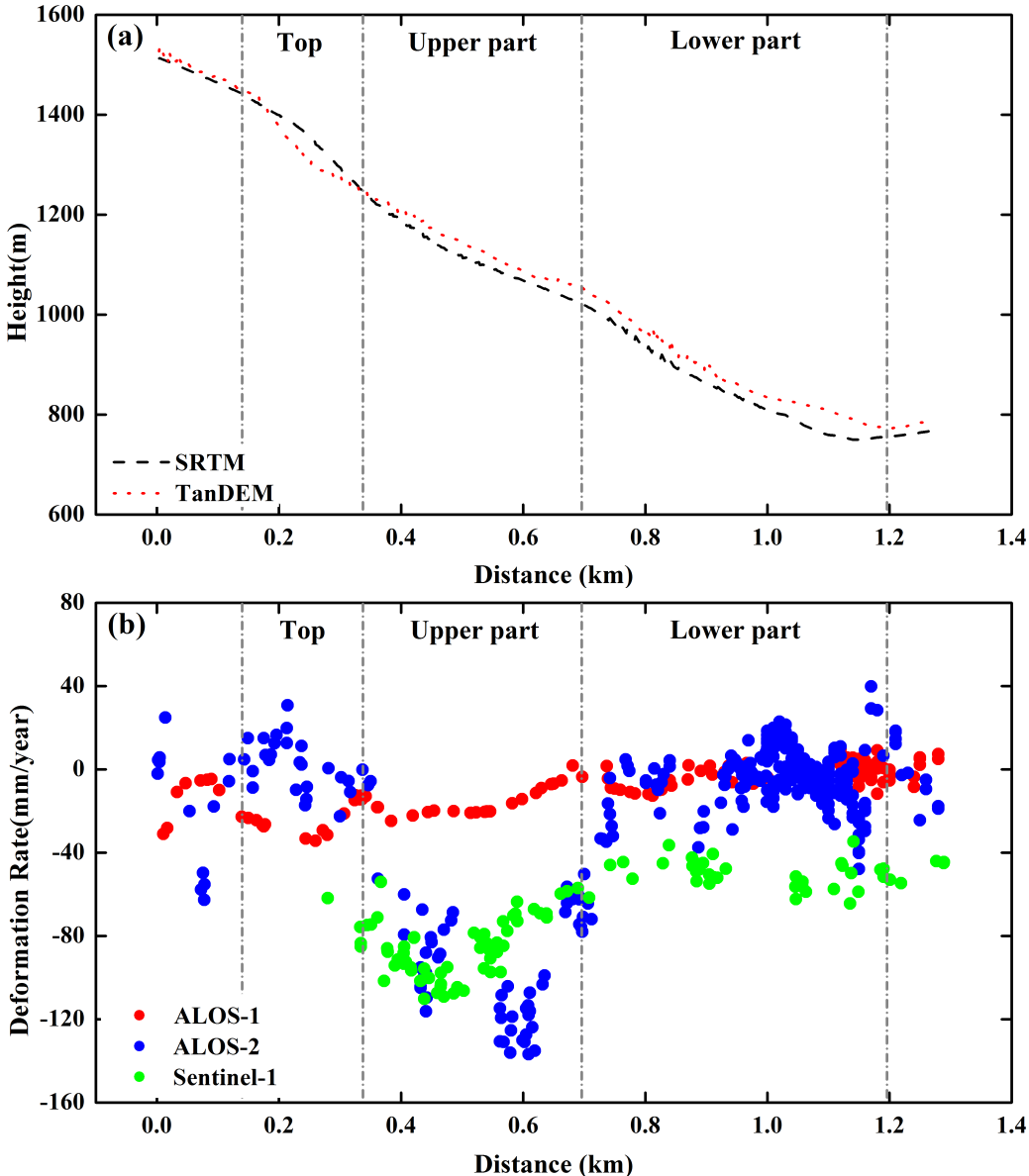


**Supplementary Fig. S4**. Plots of (a) elevations of SRTM and TanDEM DEM and (b) pre- and post-disaster deformation rates along profile I-I’.

The mean deformation rates over pre- and post-disaster stages together with the elevations of SRTM and post-disaster TanDEM are extracted along the profile I-I’ (marked in Fig. 7 (a)) and plotted in Supplementary Fig. S4 online. By joint visualization of elevation and deformation measurements, we can see that the pre-disaster active deformation was mainly concentrated in the upper part of the landslide, coinciding with the depletion zone where the elevation was decreased drastically as the result of the collapse.

Most debris detached from the source area was accumulated at the slope toe, blocking the Sunkoshi River to form a dam and a barrier lake. The elevation change in the accumulation zone was identified as wide-spreading and slight to moderate increase. The post-disaster deformation detected in the accumulation area was mainly in the upper part, while other parts of this area were relatively stable. However, as shown in the Sentinel-1 InSAR result (Fig. 7 (b)), clear signs of instability can be identified around the reopened highway.

In contrast, a large volume of debris was accumulated on the resistant layer in the upper part of the landslide. Since such debris was loosely structured without any stabilization, this elevated area could be in high risk of catastrophic sliding in cases of earthquake or heavy rainfall, which may pose great threats upon human and vehicles passing through the highway. Therefore, it is vital to keep a close eye on the long-term stability of the landslide.


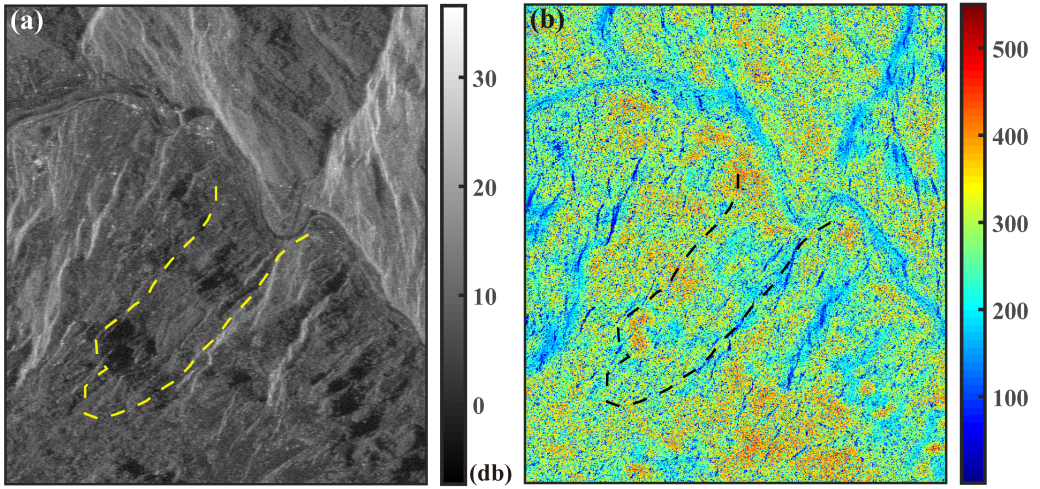


**Supplementary Fig. S5**. (a) Multi-image mean intensity map of ALOS PALSAR and (b) number of SHPs identified by K-S test in radar coordinate system. The dashed line outlines the landslide boundary.


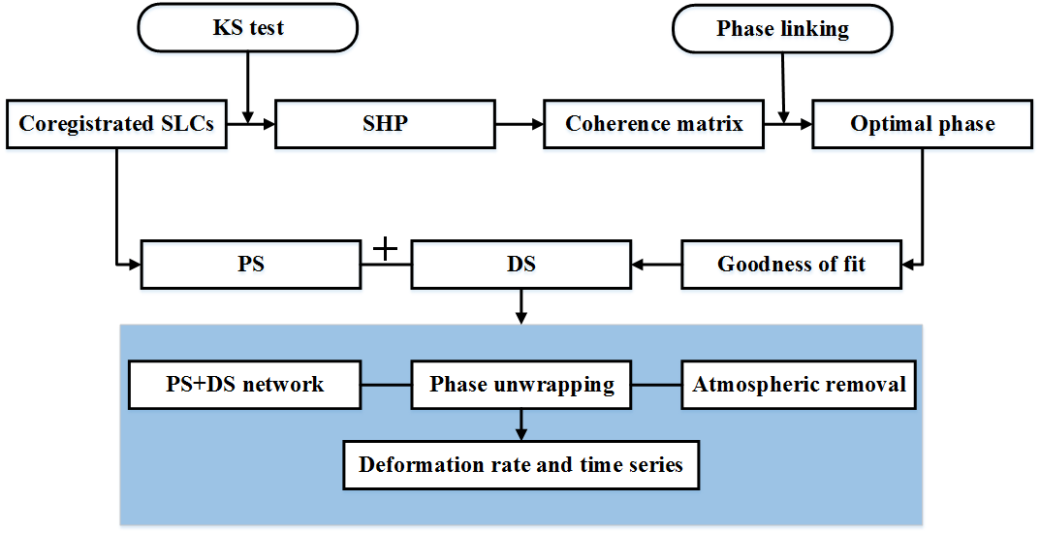


**Supplementary Fig. S6**. Flowchart of DS-InSAR processing.


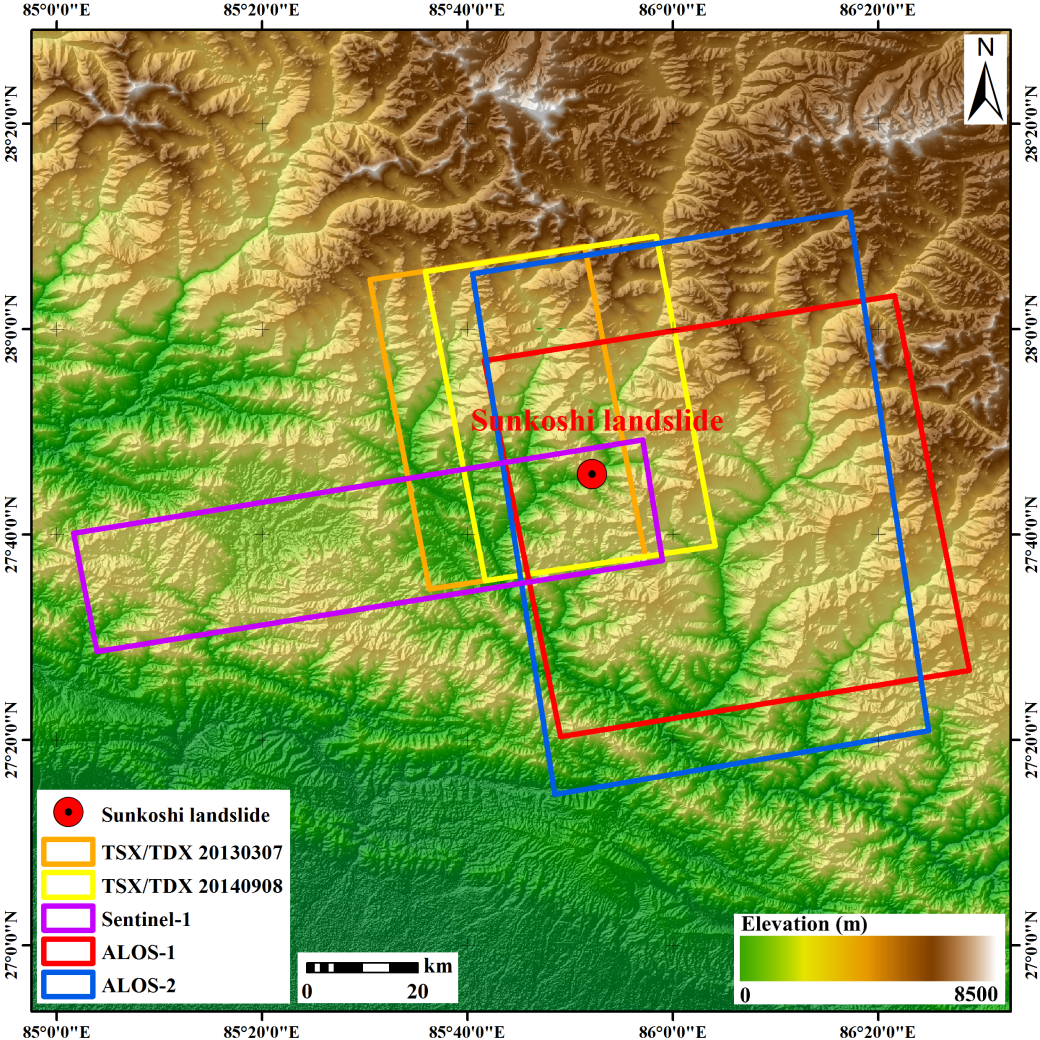


**Supplementary Fig. S7**. Ground coverages of four groups of satellite SAR datasets. The map was generated using ESRI ArcGIS 10.1 (<https://www.esri.com/en-us/home>).





**Supplementary Fig. S8**. Spatial-temporal baseline distributions of SAR datasets. The left y-axis is applicable exclusively to ALOS PALSAR data, while the right y-axis of much smaller scale serves for other datasets including TSX/TDX, ALOS-2 PALSAR-2 and Sentinel-1.

**Supplementary Table S1**. Acquisition parameters of all SAR datasets

| Parameters | ALOS PALSAR | ALOS-2 PALSAR-2 | Sentinel-1 | TSX/TDX | TSX/TDX |
| --- | --- | --- | --- | --- | --- |
| Temporal coverage | **20070113-20110124** | **20140906-20171028** | **20170104-20191021** | **20130307** | **20140908** |
| No. of images | **20** | **11** | **82** | **2** | **2** |
| Orbit direction | **Ascending** | **Ascending** | **Ascending** | **Ascending** | **Ascending** |
| Polarization | **HH & HH/HV** | **HH/HV** | **VV** | **HH** | **HH** |
| Incidence angle (degree) | **38.7** | **40.556** | **39.332** | **43.06/43.12** | **42.29/42.33** |
| Heading (degree) | **347.41** | **344** | **347.402** | **350.23** | **350.18** |
| Range/Azimuth  resolution (m) | **4.68/3.52** | **4.29/3.26** | **2.33/13.96** | **2.57/3.30** | **2.61/3.30** |
